# Obstructive Sleep Apnea: Risk Factor for Arrhythmias, Conduction Disorders, and Cardiac Arrest

**DOI:** 10.7759/cureus.9992

**Published:** 2020-08-24

**Authors:** Roshan Acharya, Sijan Basnet, Biswaraj Tharu, Ajay Koirala, Rashmi Dhital, Pragya Shrestha, DilliRam Poudel, Sushil Ghimire, Smita Kafle

**Affiliations:** 1 Internal Medicine, Cape Fear Valley Medical Center, Fayetteville, USA; 2 Internal Medicine, Campbell University School of Osteopathic Medicine, Fayetteville, USA; 3 Internal Medicine, The Reading Hospital and Medical Center, Reading, USA; 4 Internal Medicine, Western Reserve Health Education/Northeast Ohio Medical University, Youngstown, USA; 5 Rheumatology, Hospital of Philadelphia, Philadelphia, USA; 6 Hematology, Thomas Jefferson University, Philadelphia, USA; 7 Nursing, Fayetteville State University, Fayetteville, USA

**Keywords:** obstructive sleep apnea, arrhythmia, conduction disorder, mortality, national inpatient sample

## Abstract

Background

Obstructive sleep apnea (OSA) has been described as a risk factor for cardiac arrhythmias. Its association with atrial fibrillation has been established. However, relationships with other arrhythmias and conduction disorders have not been fully studied.

Methods

We used the National Inpatient Sample database from 2009 to 2011 to explore the relationship between OSA and arrhythmias and conduction disorders. The presence of diagnosis was determined based on the International Classification of Disease-9 (ICD-9) codes. Univariate and multivariate logistic regressions were used to establish mortality risks among all groups.

Results

Multivariate logistic regression showed increased mortality in patients with OSA in comparison to patients without OSA and patients across all categories of arrhythmias and conduction disorders. One significant finding was the increased association of cardiac arrest in patients with OSA versus patients without OSA (OR: 95.72; CI: 89.13-105.81, p < 0.001).

Conclusions

OSA is significantly associated with non-atrial fibrillation arrhythmias, conduction disorders, and sudden cardiac arrest. Awareness regarding this association is important for early screening for OSA in obese patients to prevent cardiovascular morbidity and mortality. The use of continuous positive airway pressure (CPAP) might be beneficial against all kinds of arrhythmias and sudden cardiac death.

## Introduction

In the United States, 35.5% of adult men and 35.8% of adult women are obese [1]. The obesity epidemic has led to an increased prevalence of obstructive sleep apnea (OSA) [2]. In the Wisconsin sleep cohort study, the prevalence of OSA was estimated to be 2% among women and 4% among men [3]. Lee et al. estimated that up to 80% of moderate or severe cases of OSA are undiagnosed despite access to healthcare [2]. OSA is being increasingly identified as a cardiovascular risk factor and has been associated with heart failure, coronary artery disease, hypertension, and arrhythmias [2,4].

Except for atrial fibrillation, the risk of arrhythmias with OSA has not been fully established [5,6]. Cintra et al. studied 767 volunteers, including both controls and OSA patients, for cardiac arrhythmias. They observed a significant difference in the occurrence of cardiac arrhythmias like premature ventricular complexes, ventricular bigeminy, atrial premature complex, chronic and paroxysmal atrial fibrillation, and non-sustained supraventricular and ventricular tachycardias among the two groups. However, no significant differences were observed in ventricular fibrillation, complete heart block, or cardiac arrest among patients with or without OSA [7]. Studies done to explore the association between OSA and arrhythmias were unsuccessful in finding the incidence of significant disorders such as ventricular arrhythmias and heart block with OSA [8]. However, they were done with small sample sizes and were poorly designed [5,9]. Moreover, the evidence linking OSA with mortality in patients with arrhythmias is limited and inconclusive [5,8,10].

In our study using a US national database, we try to explore the possible association of mortality in hospitalized patients with OSA and significant rhythm disorders such as atrial fibrillation, ventricular fibrillation, complete heart block, and cardiac arrest.

## Materials and methods

Study population

We utilized the National Inpatient Sample (NIS) database from the years 2009 to 2011. NIS is the largest all-payer inpatient care database in the US and contains discharge level information of 7-8 million hospital stays. This is a 20% stratified clustered sample from all US hospitals. It is federally sponsored by the Agency for Healthcare Research and Quality [11,12]. Patients (≥18 years) admitted to US hospitals from 2009 to 2011 were selected. Patients with OSA were selected based on the International Classification of Disease-9 (ICD-9) code 327.23. OSA was analyzed in relation to arrhythmias, atrial fibrillation, ventricular fibrillation, cardiac arrest, conduction disorders, and complete heart block using their respective ICD-9 codes. Data for baseline characteristics such as age, gender, race, expected primary payer, and mean household income for the patient's ZIP code were also obtained from the same database [11,12]. The primary outcome for this study group was in-hospital mortality.

Statistical methods

STATA version 13.0 (College Station, TX) was used for the analysis of this survey sample data. Categorical data were expressed as a percentage with Chi-square testing used to compare percentages. Continuous variables were reported as means with standard errors. Associations of mortality in OSA with various arrhythmias were compared by univariate and multivariate analyses with logistic regression. Multivariate analyses were controlled for age, sex, race, smoking, obesity, dyslipidemia, diabetes, ischemic heart disease, valvular heart disease, heart failure, transient ischemic attack, stroke, hypothyroidism, chronic kidney disease, and Charlson comorbidity index. A two-tailed p-value of p < 0.05 was considered significant.

## Results

**Figure 1:**
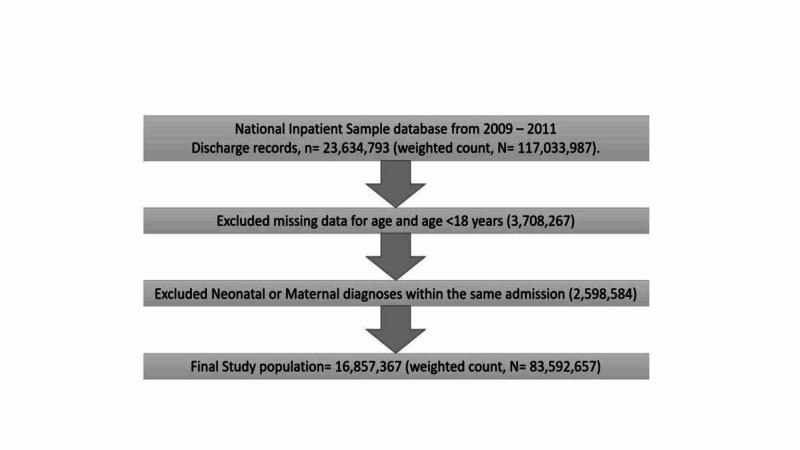
Selection process for the discharge records used in the final analysis

The total number of discharge records in the NIS database from 2009 to 2011 was 23,634,793 (weighted count in the universe of the database, N = 117,033,987). Over 16,857,367 (weighted count, N = 83,592,657) records were used for analysis after exclusion as explained in Figure 1.

The baseline characteristics of OSA patients are presented in Table 1. OSA was more common in males. OSA was more than five times frequently associated with obesity (48.14% vs 9.05%, p < 0.001). Other disorders such as diabetes mellitus, chronic kidney disease, hyperlipidemia, heart failure, valvular heart disease, hypothyroidism, and smoking were found to have a higher association with OSA. Overall, OSA patients had more associated comorbidities than non-OSA patients as shown by the higher Charlson comorbidity index which was controlled during multivariate analyses. Table 2 represents the association of mortality in OSA patients with arrhythmias and conduction disorders in comparison to non-OSA patients with arrhythmias and conduction disorders. Mortality was significantly higher across all categories of arrhythmias and conduction disorders when associated with OSA. Univariate and multivariate analyses of the association of mortality in arrhythmias and conduction disorders in patients with and without OSA are presented in Table 3. The odds of mortality in OSA patients with ventricular fibrillation in comparison to non-OSA patients was higher (OR: 22.32; 95% CI: 18.21-27.36, p < 0.001). Greater odds of mortality was seen with cardiac arrest in OSA patients in comparison to non-OSA patients (OR: 85.15; 95% CI: 76.74-94.49, p < 0.001). Although the odds of mortality in OSA patients with atrial fibrillation (OR: 1.42; 95% CI: 2.15-2.39, p < 0.001) was also increased, it was much lower compared to cardiac arrest or ventricular fibrillation.

**Table 1:** Baseline characteristics of admitted patients with and without OSA expressed in percentages. OSA: obstructive sleep apnea.

Baseline characteristics	No OSA	OSA	p-value
Age	61.70	60.91	<0.001
Female	54.04	44.11	<0.001
Race			
White	70.64	75.09	<0.001
Black	14.94	15.65	<0.001
Hispanic	8.95	5.95	<0.001
Asian or Pacific Islander	1.93	0.75	<0.001
Native American	0.65	0.55	<0.001
Expected primary payer			
Medicare	51.79	53.51	<0.001
Medicaid	11.25	9.47	<0.001
Private insurance	26.85	30.89	<0.001
Self-pay	6.04	2.91	<0.001
No charge	0.64	0.31	<0.001
Mean household income for patient’s Zipcode			
0-25^th^ percentile	29.59	27.78	<0.001
26-50^th^ percentile	25.83	26.38	<0.001
51-75^th^ percentile	24.07	25.75	<0.001
76-100^th^ percentile	20.50	20.08	<0.001
Mean Charlson comorbidity index	1.63	2.23	<0.001
Risk factors			
Obesity	9.05	48.14	<0.001
Smoking	24.50	28.58	<0.001
Diabetes mellitus	25.71	48.26	<0.001
Chronic kidney disease	13.10	20.36	<0.001
Hyperlipidemia	29.14	44.64	<0.001
Ischemic heart disease	24.23	33.83	<0.001
Transient ischemic attack	0.90	0.77	<0.001
Stroke	2.66	1.90	<0.001
Peripheral vascular disease	0.11	0.11	0.51
Heart failure	14.69	29.53	<0.001
Valvular heart disease	6.66	8.54	<0.001
Hypothyroidism	11.57	15.08	<0.001

**Table 2:** Association of mortality in patients with arrhythmias and conduction disorders in relation to the presence or absence of OSA expressed as odds ratios. OSA: obstructive sleep apnea.

Mortality	No OSA	OSA	p-value
Arrhythmias	25.21	52.26	<0.001
Atrial fibrillation	18.75	30.92	<0.001
Ventricular fibrillation	0.08	1.47	<0.001
Cardiac arrest	0.17	11.35	<0.001
Conduction disorders	0.38	0.88	<0.001
Complete heart block	0.35	0.84	<0.001

**Table 3:** Univariate and multivariate analyses with logistic regression of mortality of OSA. OSA: obstructive sleep apnea. *Adjusted for age, sex, race, smoking, obesity, dyslipidemia, chronic kidney disease, hypertension, heart failure, ischemic heart disease, and valvular heart disease.

Mortality in OSA patients	Unadjusted odds ratio	Adjusted*odds ratio
Arrhythmias	3.24 (95% CI: 3.11-3.39), p < 0.001	2.27 (95% CI: 2.15-2.39), p < 0.001
Atrial fibrillation	2.37 (95% CI: 2.27-2.48), p < 0.001	1.42 (95% CI: 1.35-1.50), p < 0.001
Ventricular fibrillation	25.78 (95% CI: 21.65-30.69), p < 0.001	22.32 (95% CI: 18.21-27.36), p < 0.001
Cardiac arrest	97.12 (95% CI: 89.13-105.81), p < 0.001	85.15 (95% CI: 76.74-94.49), p < 0.001
Conduction disorders	2.30 (95% CI: 1.88-2.81), p < 0.001	1.80 (95% CI: 1.45-2.21), p < 0.001
Complete heart block	2.41 (95% CI: 1.96-2.96), p < 0.001	1.84 (95% CI: 1.48-2.30), p < 0.001

## Discussion

Different mechanisms have been proposed regarding the pathogenesis of arrhythmias and conduction disorders in OSA. OSA causes repeated hypoxia/hypoxemia during episodes of apnea/hypopnea throughout sleep [9]. This results in increased production of reactive oxygen species and inflammatory markers with resulting endothelial dysfunction [9,13]. Hypoxemia and hypercapnia observed during apnea/hypopnea also lead to the activation of chemoreceptors, which in turn increases the sympathetic drive and catecholamine surge. The downstream effect is an increase in myocardial oxygen demand which coincides with a time when oxygen saturation is at a nadir. The myocardial ischemia may potentially cause fatal arrhythmias and cardiac arrest [14,15]. Voigt et al. also proposed that hypoxemia-triggered carotid body stimulation increases the vagal tone to induce bradyarrhythmias. They proposed that it was related to increased vagal tone as it resolved after IV atropine and/or supplemental oxygen [16]. Abe et al. suspect that there may be fluctuations in the autonomic tone in OSA with increased susceptibility to different types of arrhythmias [17]. Aytemir et al. suggest that parasympathetic activity is also decreased in OSA resulting in unopposed sympathetic activity, in-turn resulting in a shortened refractory period of the ventricular myocardium, increasing the risk of ventricular arrhythmias [18]. Moreover, the repeated hypoxia/hypoxemia episodes in OSA may lead to ventricular ectopy, predisposing to ventricular arrhythmias [15]. Roche et al. followed 147 consecutive patients who underwent polysomnogram for suspected OSA and noticed an increased association of OSA with premature ventricular complexes but not with other ventricular arrhythmias [5]. Our study was able to find significantly increased mortality with ventricular fibrillation in OSA patients than those without OSA (Table 2).

Negative intrathoracic pressure generated during apnea/hypopnea in OSA has also been described in the pathogenesis of arrhythmias by increasing direct tension on the intrathoracic structures such as the heart. This was found to shorten the atrial refractory period and enhance single premature beats to induce atrial fibrillation [19,20]. Gami et al. described obesity and OSA as an independent risk factor for atrial fibrillation in patients less than 65 years [21]. Gami et al., in another retrospective study, followed up 3542 subjects referred for a sleep study for a mean period of 4.7 years and found incident atrial fibrillation in 133 subjects (cumulative probability: 14%; 95% CI: 9-19%). The association of sleep apnea with mortality was not studied [6]. Our study found a similar but slightly greater incidence of atrial fibrillation with OSA (Table 2). Abe et al. found a significant association of OSA with paroxysmal atrial fibrillation (adjusted odds ratio: 6.44; 95% CI, 1.53-27.08, p = 0.011). However, they did not find any difference in premature atrial complex, sinus bradycardia, and second-degree atrioventricular (AV) block with or without OSA [17].

Negative intrathoracic pressure can also be simulated by the Muller maneuver and inspiratory threshold device. The tension generated by fluctuations in intrathoracic pressure in OSA may also cause QT-prolongation with an effect on cardiac repolarization, leading to an increased sympathetic activation. Prolonged QT interval is another risk factor for fatal arrhythmias such as torsades de pointes leading to cardiac arrest [19]. Gami et al. in a study of 10,701 consecutive patients referred for a sleep study for a mean duration of 5.3 years and concluded nocturnal hypoxemia and lowest nocturnal oxygen saturation as predictors of sudden cardiac death [15]. In our study, we found increased mortality in OSA patients with ventricular fibrillation in comparison to non-OSA patients.

Roche et al. noticed a greater incidence of nocturnal sinus bradycardia and sinoatrial paroxysmal block in OSA but not significant conduction disorders such as complete AV block [5]. Koehler et al. followed 16 patients with moderate to severe OSA and recorded nocturnal bradyarrhythmias but no day time ones. They recorded 178 episodes of sinus arrest, 432 episodes of second-degree AV block type Mobitz, and 41 episodes of third-degree AV block. Of all arrhythmias, 56.1% were recorded below oxygen saturation of 72%. Over 609 episodes of heart block were observed with desaturation of at least 4% [22]. Becker et al. observed that episodes of the second- and third-degree block and/or sinus arrest are significantly higher in obese (mean: 38.7 (SD: 7.3) versus mean: 30.7 (SD: 4.6)) [23]. Although increased AV block was seen in OSA, the association of conduction-disorder-related mortality in OSA patients was not established [5,22,23].

Doherty et al. found a significant reduction in cardiovascular deaths (not contributed by arrhythmia) in OSA patients using continuous positive airway pressure (CPAP) in comparison to OSA patients not using CPAP (RR: 0.08; CI: 0.01-0.52, p-value 0.008) [9]. In contrast, Yu et al. in their meta-analysis of randomized controlled trials did not find any reduction in cardiovascular events despite the use of CPAP [24]. Similar to Yu et al., McEvoy et al. did not find any benefits in their SAVE study [4]. However, both studies do not mention arrhythmias as one of the measured composite cardiovascular endpoints [4,24]. Thus, the benefit of CPAP in relation to the reduction of arrhythmias and conduction disorders is unclear. Since hypoxemia/hypercapnia is believed to be the trigger for arrhythmias and greatest risk for sudden cardiac death is during sleep from midnight to early morning, CPAP may still have some benefit against them [21,23,25].

## Conclusions

OSA patients are at greatest risk for sudden cardiac death during sleep hours; midnight to early morning. Thus, using a CPAP at night may still be beneficial against the prevention of arrhythmias, conduction disorders, and sudden cardiac arrest. It will be interesting to see studies exploring this in the future.

## Appendices

**Table 4:** ICD-9 diagnoses and associated codes ICD-9: International Classification of Diseases, Ninth Revision.

S.No.	ICD-9 diagnosis	ICD-9 code
1	Arrhythmias	427, 427.0, 427.1, 427.2, 427.3, 427.31, 427.32, 427.4, 427.41, 427.42, 427.5, 427.6, 427.60, 427.61, 427.69, 427.8, 427.81, 427.89, 427.9
2	Atrial fibrillation	427.31
3	Ventricular fibrillation	427.41
4	Cardiac arrest	427.5
5	Conduction disorders	426, 426.0, 426.1, 426.10, 426.11, 426.12, 426.13, 426.2, 426.3, 426.4, 426.5, 426.50, 426.51, 426.52, 426.53, 426.54, 426.6, 426.7, 426.8, 426.81, 426.82, 426.89, 426.9
6	Complete heart block	426.0
